# Advances in Pharmacological Treatment of Thoracic Malignancies

**DOI:** 10.14789/ejmj.JMJ25-0049-R

**Published:** 2026-03-04

**Authors:** TAKEHITO SHUKUYA, KAZUHISA TAKAHASHI

**Affiliations:** 1 Department of Respiratory Medicine, Juntendo University School of Medicine, Tokyo, Japan; 1 Department of Respiratory Medicine, Juntendo University School of Medicine, Tokyo, Japan

**Keywords:** lung cancer, targeted therapy, immunotherapy, antibody drug conjugate, bispecific T cell engager

## Abstract

Lung cancer remains the leading cause of cancer-related death worldwide, with over two million new cases annually. Advances in molecular biology and immuno-oncology have fundamentally transformed treatment strategies. Comprehensive genomic profiling has enabled precision medicine approaches, allowing the selection of targeted therapies based on driver alterations such as *EGFR*, *ALK*, and *HER2*. For *EGFR*-mutant NSCLC, sequential development of tyrosine kinase inhibitors (TKIs) from first- to third-generation agents―culminating in osimertinib―has markedly improved survival. Similarly, successive generations of ALK inhibitors, including alectinib, brigatinib, and lorlatinib, have extended disease control, particularly within the central nervous system. The introduction of antibody-drug conjugates (ADCs), such as trastuzumab deruxtecan for *HER2*-mutant NSCLC, and emerging TKIs like zongertinib, represent new therapeutic milestones.

Immunotherapy has become central to the management of both NSCLC and SCLC, with immune checkpoint inhibitors (ICIs) demonstrating unprecedented survival benefits. Beyond lung cancer, our group, in collaboration with Juntendo University ARO (academic research organization) and fifteen institutions in Japan, conducted the MARBLE phase II trial of atezolizumab plus chemotherapy for thymic carcinoma, achieving a 56% objective response rate and 9.6-month median progression-free survival, supporting potential ICI approval in Japan. Furthermore, novel immune strategies such as bispecific T-cell engagers (BiTEs) have shown promise. The DLL3-targeted BiTE tarlatamab significantly improved overall survival to 13.6 months in the phase III DeLLphi-304 trial for relapsed SCLC, with manageable cytokine release syndrome. Collectively, these advances signify a shift toward biologically driven, molecular-targeted or immune-integrated therapy, aiming to transform lung cancer into a chronic, manageable disease in the future, hopefully.

## Introduction

Lung cancer is the most common cause of cancer- related mortality worldwide, accounting for nearly 1.8 million deaths annually and more than 2.2 million new cases each year^1)^. Despite improvements in screening and early detection, the majority of patients are still diagnosed at advanced stages, where prognosis remains poor. Non-small cell lung cancer (NSCLC) comprises approximately 85% of cases, while small cell lung cancer (SCLC) accounts for 10-15%^2)^.

The incidence and molecular profiles of lung cancer vary significantly across geographic regions. For instance, epidermal growth factor receptor (EGFR) mutations are highly prevalent in East Asia (30-40% of NSCLC cases), but less common in Western populations (10-15%). Conversely, *KRAS* mutations are frequently observed in Western populations (20-30%) but are relatively rare in Asian cohorts^3, 4)^. Other driver alterations-including *ALK, ROS1, RET, MET* exon 14 skipping, *BRAF* V600E, *HER2* mutations, and *NTRK* fusions - each account for smaller subsets, typically ranging from 1% to 7% of NSCLC cases.

This molecular diversity underpins the paradigm shift toward precision oncology, where molecular testing guides therapy selection. The implementation of comprehensive next-generation sequencing (NGS) has enabled simultaneous detection of multiple actionable alterations, ensuring patients receive the most appropriate targeted therapy^2)^.

**Figure 1:**
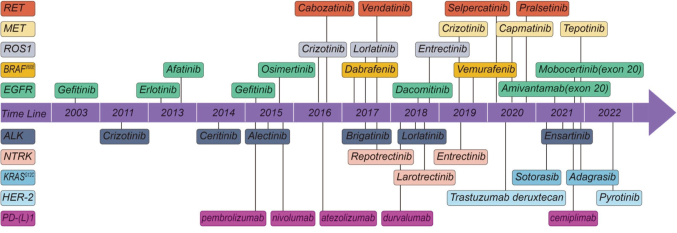
Advances in systemic therapy for NSCLC Overview of the evolution of systemic treatments for non-small cell lung cancer, highlighting the shift from cytotoxic chemotherapy to targeted and immune therapies. Adapted from Guo H, et al. Biomarker-Targeted Therapies in Non-Small Cell Lung Cancer: Current Status and Perspectives. Cells. 2022 Oct 12; 11(20): 3200.

Historically, lung cancer therapy relied on platinum-based chemotherapy, which offered limited survival benefit (median overall survival 8-12 months for advanced disease). The advent of targeted therapy-pioneered by the discovery of *EGFR* mutations and *ALK* rearrangements-has transformed NSCLC treatment. Patients with these mutations can now achieve median survivals exceeding 3-5 years with sequential tyrosine kinase inhibitors (TKIs). In parallel, immune checkpoint inhibitors (ICIs) have revolutionized the management of both NSCLC and SCLC. Anti-PD-1/PD-L1 antibodies, alone or in combination with chemotherapy, have demonstrated unprecedented survival benefits, with long-term survival plateaus emerging in selected populations^2)^. As shown in Figure 1, numerous drugs have been developed and introduced into clinical practice based on these backgrounds.

Beyond TKIs and ICIs, a new generation of therapies is being developed. These include antibody- drug conjugates (ADCs), bispecific T-cell engagers (BiTEs), chimeric antigen receptor T-cell (CAR- T) therapies, and cancer vaccines. Each modality aims to address resistance mechanisms and provide therapeutic options for patients lacking conventional targets.

In this review, we describe the development of molecular targeted agents which target representative driver oncogenes and advances in immunotherapy with the novel mechanism drugs such as bispecific antibody and ADC.

### EGFR-targeted therapy

*EGFR* mutations are the most common oncogenic drivers in NSCLC, particularly among never-smokers and East Asian patients. The two principal activating mutations-exon 19 deletions and the L858R point mutation in exon 21-account for approximately 90% of cases and lead to constitutive activation of downstream signaling pathways that promote malignant cell growth. Notably, when gefitinib was first used clinically, high activity was observed in East Asian patients, never-smokers, and those with adenocarcinoma^3)^; subsequently, somatic *EGFR* mutations were discovered and became the predictive biomarker guiding EGFR-TKI use - an episode that marked the dawn of molecularly targeted therapy in lung cancer^5, 6)^.

The introduction of EGFR-TKIs revolutionized treatment for this molecular subtype. The NEJ002 and WJTOG3405 phase III trials in Japan firmly established gefitinib as the standard first-line therapy for EGFR-mutated advanced NSCLC. In NEJ002, gefitinib achieved an objective response rate (ORR) of 73.7% vs 30.7% (p < 0.001) and median PFS of 10.8 vs 5.4 months (HR 0.30; p < 0.001) compared with carboplatin plus paclitaxel, while OS (27.7 vs 26.6 months; p = 0.48) was confounded by treatment crossover^7)^. Similarly, WJTOG3405 reported median PFS of 9.2 vs 6.3 months (HR 0.49; p < 0.001) and ORR of 62% vs 32% (p < 0.001) versus cisplatin plus docetaxel, confirming the superiority of EGFR-TKIs despite the absence of an OS difference^8)^.

The second-generation EGFR-TKI afatinib, an irreversible ErbB family blocker, further extended disease control (PFS median 11.1 vs 6.9 months; HR 0.58; p < 0.001) and demonstrated efficacy in uncommon *EGFR* mutations such as G719X, L861Q, and S768I^9)^. Toxicities including diarrhea, rash, and stomatitis were frequent but manageable with proactive supportive care and dose adjustment.

The third-generation TKI osimertinib, developed to overcome the T790M resistance mutation, became the next therapeutic milestone. In the FLAURA trial, osimertinib significantly improved PFS (median 18.9 vs 10.2 months; HR 0.46; p < 0.001) and OS (median 38.6 vs 31.8 months; HR 0.80; p = 0.046) compared with first-generation TKIs, and demonstrated excellent CNS activity (ORR 91%) in patients with brain metastases^10)^. Adverse events such as rash, diarrhea, and paronychia were common, while interstitial lung disease (ILD) (3-5%) was more frequent in Japanese patients, requiring prompt recognition and management.

Despite these advances, acquired resistance inevitably emerges. The main mechanisms of resistance include C797S mutation, MET amplification, and histologic transformation to small cell or squamous phenotypes. To address these, new therapeutic strategies are under investigation, such as fourth- generation EGFR-TKIs targeting C797S, combination regimens incorporating EGFR and MET blockade, and bispecific antibodies.

Among these, the MARIPOSA trial evaluated the bispecific EGFR/MET antibody amivantamab in combination with lazertinib, 3rd generation EGFR-TKI, versus osimertinib in treatment-naïve *EGFR*-mutated NSCLC. The combination achieved a superior ORR (86% vs 76%) and longer PFS (median 23.0 vs 16.6 months; HR 0.70; p < 0.001), with durable CNS control. The most common adverse events were infusion-related reactions (65%), rash, and paronychia, managed by pre-infusion prophylaxis and dermatologic care^11)^.

Collectively, the evolution from first- to third- generation EGFR-TKIs-and now to bispecific antibodies and combination approaches - illustrates a dynamic therapeutic landscape aimed at overcoming resistance and achieving more durable disease control in *EGFR*-mutated NSCLC.

### ALK-targeted therapy

The anaplastic lymphoma kinase (ALK) gene rearrangement, first identified in 2007 in NSCLC, occurs in about 3-7% of adenocarcinomas, typically in younger, never- or light-smokers^12)^. The most common fusion partner is *EML4*, though over 20 variants have been identified, all leading to constitutive kinase activation and oncogenesis^12)^. These alterations confer strong sensitivity to ALK-TKIs, which have transformed outcomes in this molecular subtype. Over the past decade, successive generations of ALK-TKIs have replaced chemotherapy, and many patients now achieve survival exceeding five years.

The first-generation TKI crizotinib demonstrated clear superiority over chemotherapy in the PROFILE 1014 trial, showing an ORR of 74% vs 45% (p < 0.001) and median PFS of 10.9 vs 7.0 months (HR 0.45; p < 0.001)^13)^. These results established targeted therapy as the standard of care, though limited CNS penetration and eventual resistance prompted the development of next-generation inhibitors.

The second-generation ALK-TKIs, alectinib and brigatinib, produced deeper and more durable responses with excellent CNS activity. In the ALEX trial, alectinib significantly improved PFS (median 34.8 vs 10.9 months; HR 0.43; p < 0.001) and intracranial ORR (81% vs 50%; p < 0.001) compared with crizotinib, resulting in a 5-year OS rate of 62.5%^14)^. Similarly, the ALTA-1L trial demonstrated superior efficacy for brigatinib, with median PFS of 24.0 vs 11.0 months (HR 0.49; p<0.001) and strong CNS penetration^15)^. Both agents were generally well tolerated, with manageable adverse events such as myalgia, hepatotoxicity, and early-onset pulmonary events.

The third-generation inhibitor lorlatinib, evaluated in the CROWN trial, further advanced outcomes, showing an ORR of 76% vs 58% (p < 0.001), 3-year PFS of 64% vs 19% (HR 0.27; p < 0.001), and intracranial ORR of 82% compared with crizotinib^16)^. Lorlatinib also demonstrated potent activity against resistance mutations, particularly G1202R, which are refractory to earlier TKIs. The main toxicities included hyperlipidemia, CNS effects, edema, and weight gain, managed through statins, psychiatric monitoring, and dose adjustments.

Despite these major advances, acquired resistance remains inevitable, commonly driven by secondary ALK mutations or bypass signal pathway activation. Ongoing research focuses on fourth-generation ALK inhibitors and combination strategies to overcome resistance and prolong disease control.

In clinical practice, comprehensive genomic profiling is essential for accurate diagnosis, and CNS surveillance remains critical given the high risk of brain metastases. Overall, ALK-positive NSCLC exemplifies the success of precision oncology, where progressive generations of ALK-TKIs have prolonged survival, improved quality of life, and enabled durable long-term disease control through careful sequential therapy and adverse event management.

### HER2-targeted therapy, advent of ADC

HER2 (ERBB2) alterations occur in approximately 2-4% of NSCLC. The most common alterations are exon 20 insertions (around 2%), followed by point mutations such as L755P and V777L. HER2 amplification and overexpression are less frequent in NSCLC than in breast or gastric cancer^17, 18)^. Unlike *EGFR* or *ALK*, *HER2* mutations long lacked effective targeted therapies. Early HER2-TKIs such as afatinib, neratinib, and poziotinib demonstrated limited efficacy and significant toxicity, underscoring the need for more potent and tolerable treatments.

The advent of ADCs revolutionized the treatment landscape, particularly with trastuzumab deruxtecan (T-DXd, DS-8201). In the phase II DESTINY- Lung01 trial, previously treated patients with *HER2*- mutant NSCLC achieved an ORR of 55%, a median PFS of 8.2 months, and a median OS of 17.8 months^19)^. In the phase IIb DESTINY-Lung02 trial, which compared doses of 5.4 mg/kg and 6.4 mg/kg, the 5.4 mg/kg cohort achieved robust antitumor activity with favorable safety, supporting the 5.4 mg/kg dose as preferred^20)^. These results led to regulatory approval of T-DXd, establishing it as the first effective targeted therapy for *HER2*-mutant NSCLC^21)^.

The major toxicity associated with T-DXd is ILD or pneumonitis, occurring in approximately 10-15% of patients (with ~2% grade ≥ 3 in trial reports), alongside nausea, vomiting, alopecia, and cytopenias^20)^. Management strategies emphasize close monitoring with baseline and serial chest CT scans every 6-8 weeks, immediate drug interruption for any pulmonary symptoms, corticosteroid treatment for grade ≥ 2 ILD, and prophylactic antiemetic therapy with a 5-HT3 antagonist and dexamethasone.

Zongertinib (BI 1810631) has recently emerged as a next-generation, highly selective, irreversible *HER2*-TKI with promising early clinical activity in *HER2*-mutant NSCLC, particularly exon 20 insertions; as an oral agent, it may serve as an alternative or sequential option after ADCs as data mature^22)^.

### Immunotherapy to lung cancer

The introduction of ICIs targeting PD-1, PD-L1, and CTLA-4 has transformed lung cancer therapy by reactivating cytotoxic T-cell activity against tumors, achieving durable responses in subsets of patients and replacing chemotherapy as the cornerstone of treatment.

In NSCLC, KEYNOTE-024 established pembrolizumab monotherapy as the first-line standard for PD-L1 ≥ 50%. Median OS was 26.3 vs 13.4 months (HR 0.62; p = 0.001), with a 5-year OS rate of 31.9% vs 16.3%^23, 24)^. In combination settings, KEYNOTE-189 (non-squamous NSCLC) demonstrated that pembrolizumab plus platinum-based chemotherapy improved OS (median 22.0 vs 10.7 months; HR 0.56; p < 0.00001), while KEYNOTE-407 (squamous NSCLC) showed improved OS (median 17.1 vs 11.6 months; HR 0.71; p = 0.0008)^25-28)^. IMpower150 (atezolizumab + bevacizumab + chemo) reported improved OS versus bevacizumab + chemo, including efficacy in EGFR/ALK-positive post-TKI patients^29, 30)^.

For locally advanced disease, the PACIFIC trial established durvalumab consolidation after chemoradiation as the new standard for unresectable stage III NSCLC, improving OS (median 47.5 vs 29.1 months; HR 0.72; p = 0.0025)^31, 32)^. In SCLC, the IMpower133 trial (atezolizumab + chemotherapy) achieved an improved OS (median 12.3 vs 10.3 months; HR 0.70; p = 0.007), and CASPIAN (durvalumab + chemotherapy) showed median OS of 12.9 vs 10.5 months (HR 0.75; p = 0.0032), marking the first OS improvements in extensive stage SCLC in decades^33-36)^.

ICIs cause immune-related adverse events (irAEs) such as rash, thyroid dysfunction, pneumonitis, colitis, and hepatitis. Management involves corticosteroids (0.5-2 mg/kg) and treatment discontinuation for severe cases; endocrine toxicities often require lifelong hormone replacement. Early recognition is vital to prevent life-threatening complications.

PD-L1 expression remains the main biomarker, though tumor mutational burden and immune signatures are under study. Dual checkpoint blockade (nivolumab + ipilimumab) benefits select patients but increases toxicity.

Overall, immunotherapy has revolutionized lung cancer treatment. Trials like KEYNOTE-024, KEYNOTE-189, PACIFIC, and IMpower133 established ICIs as foundational across NSCLC and SCLC, delivering meaningful survival gains when guided by biomarkers and managed with vigilant toxicity control.

### Immunotherapy to thymic carcinoma

Immunotherapy has recently become an important area of investigation in thymic carcinoma, a rare and aggressive cancer with limited treatment options. Conventional platinum-based chemotherapy, such as carboplatin plus paclitaxel, has shown only modest efficacy and short-lived responses. To improve outcomes and expand the clinical application of ICIs to thymic carcinoma in Japan, our group, in collaboration with the Academic Research Organization (ARO) of Juntendo University, conducted a multicenter, single-arm, phase II trial-the MARBLE study-to evaluate the efficacy and safety of atezolizumab in combination with standard chemotherapy^37)^.

**Figure 2:**
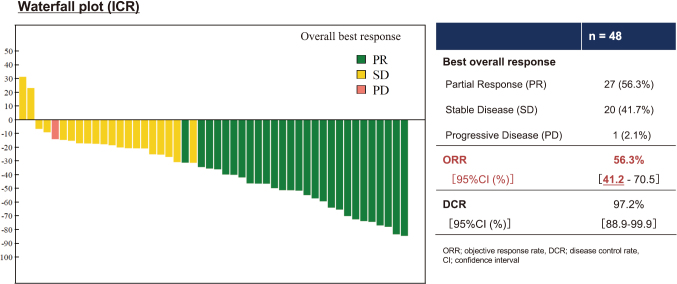
Objective response and disease control rates in MARBLE study Waterfall plot showing the best overall tumor response to carboplatin, paclitaxel plus atezolizumab in patients with thymic carcinoma. ORR and DCR are shown with 95% confidence intervals. Adapted from Goto Y, et al. The 66th Annual Meeting of The Japan Lung Cancer Society, Nov 6 to 8, 2025.

**Figure 3:**
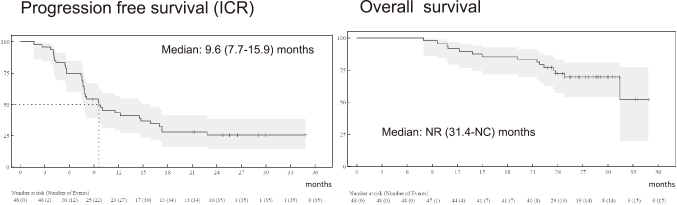
Survival outcomes in MARBLE study Kaplan-Meier curves for progression-free and overall survival. Median follow-up was 15.3 months (IQR, 13.8-16.6). Median progression-free survival and overall survival were 9.6 months and not reached, respectively. Adapted from Goto Y, et al. The 66th Annual Meeting of The Japan Lung Cancer Society, Nov 6 to 8, 2025.

In this trial, patients with previously untreated, advanced, or recurrent thymic carcinoma received atezolizumab (1200 mg) plus carboplatin (AUC 6) and paclitaxel (200 mg/m^2^) every three weeks for up to six cycles, followed by maintenance atezolizumab. A total of 48 patients were enrolled, with a median follow-up of 15.3 months. The ORR was 56% (95% CI 41-71; p < 0.0001), and the disease control rate was 98% (95% CI 89-100) (Figure 2). The median PFS was 9.6 months (95% CI 7.7-14.8)-a clear improvement compared with historical data from chemotherapy alone (Figure 3)^37)^.

Treatment-related toxicities were manageable: grade ≥ 3 events occurred in approximately 75% of patients, mainly neutropenia and leukopenia, while no treatment-related deaths were reported. IrAEs were infrequent and reversible with corticosteroid therapy.

These findings demonstrated meaningful and durable activity of atezolizumab plus chemotherapy in thymic carcinoma. The study provided the first strong evidence supporting the incorporation of immunotherapy into the frontline management of this rare malignancy and laid the groundwork for future efforts to achieve regulatory approval of ICIs for thymic carcinoma in Japan.

### Introduction of BiTE for small cell lung cancer

Bispecific T-cell engagers (BiTEs) represent a new generation of immunotherapeutic agents that redirect the body’s immune system against cancer cells. These engineered antibodies bind simultaneously to a tumor-associated antigen-such as DLL3, EGFR, or HER2-and to CD3 on T cells, thereby activating cytotoxic lymphocytes to attack tumor cells independent of MHC recognition.

Among BiTEs under development, tarlatamab (AMG 757) is a first-in-class, DLL3-directed agent that has shown remarkable activity in SCLC, a disease long characterized by limited therapeutic progress. DLL3 is selectively expressed on SCLC cells and absent from normal tissues, providing a highly specific target.

In the phase II DeLLphi-301 trial, tarlatamab achieved an ORR of 23% in heavily pretreated SCLC, with a median PFS of 3.7 months. Importantly, a plateau in survival curves suggested durable benefit in a subset of patients-an unprecedented observation in relapsed SCLC^38)^.

Building on these results, the phase III DeLLphi-304 trial compared tarlatamab monotherapy with standard chemotherapy (topotecan, lurbinectedin, or amrubicin) in patients who had progressed after platinum-based therapy. Among 509 patients, tarlatamab significantly improved OS to 13.6 months vs 8.3 months (HR 0.60; p < 0.001) and prolonged PFS (median 4.2 vs 3.7 months; HR 0.71; p < 0.001). Patient-reported outcomes also showed greater relief of respiratory symptoms and improved quality of life^39)^.

Adverse events were generally manageable. Cytokine release syndrome (CRS) occurred in approximately half of patients, predominantly grade 1-2, and neurotoxicity (ICANS) was rare. CRS was effectively controlled with step-up dosing and tocilizumab ± steroids, without fatal events^40)^. Together, these findings establish tarlatamab as a major therapeutic advance for relapsed SCLC, offering a meaningful survival advantage and manageable safety profile.

## Conclusion

Recent advances in targeted therapy, antibody- drug conjugates, and immunotherapy have dramatically improved outcomes in thoracic malignancies. These therapeutic strategies are now being explored not only in advanced disease but also in the perioperative setting, aiming to improve survival outcome. With the continuous evolution of biomedical science, further improvement in the prognosis of thoracic malignancy is anticipated. To achieve this, it is essential to maintain a strong commitment to basic, translational, and clinical research, fostering seamless collaboration between laboratory discoveries and clinical application. Through these efforts, we believe that we will be able to transform lung cancer into a chronic, manageable disease in the future with hope.

## Author contributions

TS reviewed the relevant literature and drafted the manuscript. KT supervised TS’s work and critically reviewed and revised the manuscript. All authors read and approved the final version of the manuscript.

## Conflicts of interest statement

TS receives grants from AstraZeneca, Chugai Pharmaceutical, Boehringer Ingelheim, Novartis, MSD, Novocure, Takeda Pharmaceuticals, Pfizer, and honoraria from AstraZeneca, Chugai Pharmaceutical, Boehringer Ingelheim, Novartis, MSD, Taiho Pharma, Daiichi-Sankyo, Ono Pharmaceutical, Bristol-Myers Squibb, Nippon Kayaku, Pfizer, Takeda Pharmaceuticals, Eli Lilly and Company, Eisai, Merck biopharma, Amgen. KT receives grants and honoraria from Eli Lilly Japan K. K., TAIHO PHARMACEUTICAL CO., LTD., and Nippon Kayaku Co., Ltd. KT serves on the board of directors for the Japan Lung Cancer Society and the Japanese Respiratory Society. KT is a member of the JMJ editorial board and is not involved in the peer review or decision-making process for this paper.
